# Integrating Network Pharmacology and Experimental Validation to Identifying Key Herbal Components and Targets for Liver Cancer

**DOI:** 10.5812/ijpr-162305

**Published:** 2025-09-03

**Authors:** Fang Wang, Shenghao Li, Xiling Liu, Yi Xu, Huimin Yan

**Affiliations:** 1 Hebei Medical University, Shijiazhuang, China; 2 Shanxi Technology and Business University, Taiyuan, China; 3 The Fifth Hospital of Shijiazhuang, Hebei Key Laboratory of Immune Mechanism of Major Infectious Diseases and New Technology of Diagnosis and Treatment, Shijiazhuang, China

**Keywords:** AP-1, Heat-Clearing, Invigorating qi, Liver Cancer, Traditional Chinese Medicine

## Abstract

**Background:**

Traditional Chinese medicines (TCMs) offer a comprehensive approach to managing malignant tumors.

**Objectives:**

The present study aims to predict the high-frequency herbs, core components, and core targets for liver cancer treatment through data mining and network pharmacology.

**Methods:**

The "traditional Chinese Medicine - active components - target - disease" network was established using Cytoscape to identify core components. A protein-protein interaction (PPI) network was constructed using the STRING database, and Cytoscape was used for network topological analysis to identify the core targets. Subsequently, molecular docking was performed using AutoDock Vina and PyMOL to calculate binding energies of core components with core targets. Furthermore, in vitro experiments explored quercetin’s impact on liver cancer cell migration, apoptosis, and protein expression.

**Results:**

A total of 50 high-frequency drugs were selected. Among these, *Atractylodes macrocephala* koidz, *Astragalus membranaceus*, *Scutellaria barbata*, and *Cremastra appendiculata* were high-frequency drugs for invigorating qi and heat-clearing in liver cancer treatment. There were 226 common targets of herbal medicine for liver cancer treatment. Based on the degree value, beta-sitosterol, kaempferol, stigmasterol, and luteolin potentially represented core components. Eight key targets, including JUN, MAPK1, RELA, TNF, ESR1, IL-6, TP53, and FOS, were screened out, which were involved in 417 entries and 159 pathways. Molecular docking verified a strong binding affinity of the key compounds to the core targets. In vitro experiments showed that quercetin induced apoptosis and inhibited migration activity of HepG2 cells in a dose-dependent manner by affecting the expression levels of p-c-Jun/c-Jun and c-Fos proteins.

**Conclusions:**

This study provides a foundational basis for future clinical application of TCMs in liver cancer treatment.

## 1. Background

Liver cancer is a prevalent malignancy that ranks sixth globally in cancer-related incidence and fourth in mortality rates, with the highest incidence in China (1). It is characterized by rapid progression, complex conditions, poor prognosis, high recurrence rate, and low 5-year survival rate (2). To date, common treatments for liver cancer include radiofrequency ablation, resection, transplantation, and radioactive embolism (3). However, the rate of intrahepatic spread and recurrence remains high after 5 years. Hence, it is necessary to explore effective adjuvant approaches to further reduce the progression of liver cancer.

Traditional Chinese medicines (TCMs) have demonstrated significant therapeutic value in long-term cancer treatment practices, with multi-target, multi-effect, and low toxicity. They can reduce tumor recurrence and metastasis, improve immunity and quality of life, and prolong the survival of patients (4). According to several clinical studies, the application of invigorating qi drugs to support the righteousness and heat-clearing drugs to eliminate the evil can effectively inhibit the rate of liver cancer progression to a certain extent (5-8). Currently, the primary focus of TCM treatment for liver cancer is to complement Western medicine treatment, enhancing systemic well-being and patient quality of life (9). Therefore, it is necessary to systematically summarize the rules of medication, identify high-frequency drugs, and explore their underlying mechanisms.

Activator protein-1 (AP-1), a drug therapeutic target of wide interest, functions as a key transcription factor in proliferation, differentiation, inflammation, and apoptosis through multiple pathways (10). Additionally, the AP-1 transcription factor, which is essential for MMP-9 expression, has been proven to play an important role in cellular metastasis. Hong et al. (11) showed that inhibiting the expression of c-Fos and c-Jun would block the secretion of MMP-9, thereby inhibiting cell migration and invasion. Hence, the AP-1 pathway is critical for the treatment of liver cancer.

## 2. Objectives

The present study aimed to summarize the usage patterns of TCMs against liver cancer in recent years using data mining, and explore how combining high-frequency TCMs could enhance liver cancer therapy using network pharmacology and molecular docking, to provide a theoretical basis for the combined treatment of liver cancer with TCMs.

## 3. Methods

### 3.1. Data Sources and Retrieval Strategy

The relevant literature on liver cancer treatment with TCMs in CNKI (http://www.cnki.net/) was searched from 2002 to 2022 using keywords like "liver cancer", "hepatoma", "liver tumor", "traditional Chinese medicine", "Chinese medicine", or "Chinese medicine therapy". The literature that met the requirements was extracted to form a database.

### 3.2. Inclusion and Exclusion Criteria

The inclusion criteria were as follows: (1) Diagnoses of liver cancer adhered to the "Standard for the Treatment of Primary Liver Cancer"; (2) herbal prescriptions with well-defined compositions and explicit therapeutic effects were employed in the literature; (3) the method of administration in the literature was oral; (4) clinical trials, clinical observations, and retrospective studies assessing the therapeutic effects of herbal medicines were utilized. The exclusion criteria were: (1) Unclear diagnosis; (2) medical history of remaining malignant tumors or combination of other lesions; (3) uncertain efficacy of herbal medicine; (4) repeated use of the same prescription across different literature; (5) incomplete specific medication of prescriptions or only single medication; (6) other Chinese medicine treatments such as tui na and acupuncture; (7) non-clinical studies such as animal experiments, reviews, and meta-analyses.

### 3.3. Data Standardization and Analysis

To resolve the problem of multiple names for one medicine, the TCM names from the literature were unified to official names according to the “Pharmacopoeia of the People’s Republic of China”, 2020. The standardized data was integrated into the Traditional Chinese Medicine Inheritance Assistance Platform V2.0. Subsequently, drug frequency, high-frequency drug, and efficacy classification were analyzed.

### 3.4. Prediction for Active Components and Potential Targets for Herbs

The active components and targets of *Atractylodes macrocephala* koidz, *Astragalus membranaceus*, *Scutellaria barbata*, and *Cremastra appendiculata* were predicted in the Traditional Chinese Medicine Systems Pharmacology (TCMSP) database (https://old.tcmsp-e.com/tcmsp.php). Then the obtained targets were imported into the UniProt (https://www.uniprot.org/) database for genetic standardization.

### 3.5. Screening of Liver Cancer Targets

To obtain relevant targets, disease-related terms such as “liver cancer” and “hepatocarcinoma” were employed as search keywords in electronic databases, including GeneCards (https://www.genecards.org/), OMIM (http://www.omim.org/), TTD (http://db.idrblab.net/ttd/), and DisGeNET (https://www.disgenet.org/) databases. After merging results and eliminating duplicates, liver cancer targets were extracted. Then the intersection targets were obtained by mapping them to drug targets through Venny2.1 (https://bioinfogp.cnb.csic.es/tools/venny/index.html).

### 3.6. Construction of "Traditional Chinese Medicine-Active Components-Target-Disease" Network

Along with components, the overlapping targets were imported into Cytoscape 3.9.0 (https://cytoscape.org) software to construct the "traditional Chinese medicine-active components-target-disease" network and calculate the topology parameter of the nodes in the network. Then the core components of *A. macrocephala* koidz, *A. membranaceus*, *S. barbata*, and *C. appendiculata* in the treatment of liver cancer were determined based on node degree values, MCC, EPC, and Betweenness algorithms in Cytoscape software. To systematically evaluate the pharmacokinetic and toxicological properties of the selected compounds, we employed two online platforms, SwissADME and pkCSM, to comprehensively predict and analyze their absorption, distribution, metabolism, excretion, and toxicity profiles.

### 3.7. Protein-Protein Interaction Network Construction

The PPI network was constructed using the STRING database (https://string-db.org/), and MCC, MNC, Degree, EPC, Closeness, and Betweenness algorithms in Cytoscape software were applied to network topological analysis to obtain the key targets of *A. macrocephala* koidz, *A. membranaceus*, *S. barbata*, and *C. appendiculata* against liver cancer.

### 3.8. Gene Ontology Enrichment and Kyoto Encyclopedia of Genes and Genomes Pathway Enrichment Analysis 

Gene ontology (GO) term and Kyoto Encyclopedia of Genes and Genome (KEGG) pathway enrichment were conducted through the Metascape database (http://metascape.org/) and visualized using the Bioinformatics online platform (http://www.bioinformatics.com.cn/). A "target-pathway" network was constructed to explore potential mechanisms.

### 3.9. Molecular Docking for Core Components with Core Targets

AutoDock Vina (http://vina.scripps.edu/) and PyMOL (https://pymol.org/2/) were used to dock core components with core targets to verify their binding energy. Conformations with lower binding energy and more stable docking stability were chosen for visualization and presentation.

### 3.10. Cell Culture

HepG2 cells were purchased from Procell Life Science & Technology Co., Ltd. (Wuhan, China). Cells were cultured in MEM medium supplemented with 10% fetal bovine serum (FBS, PAN-Biotech, Germany) and 100 U/mL penicillin-streptomycin (BI, Israel), maintained at 37°C in a humidified incubator with 5% CO_2_.

### 3.11. Transwell Migration Assay

First, cells were seeded in 6-well plates. After 24 hours of incubation, the lower chambers of the 24-well Transwell assay plates were filled with medium, and 100 µL of cell suspension was added to each well of the upper chamber and incubated for 12 hours. Then cells that had migrated to the lower side of the membrane were fixed and stained with 0.1% crystal violet. Several random fields were then captured under a microscope, and these pictures were used to count the total number of migrated cells. The same experiment was repeated three times.

### 3.12. Flow Cytometry Analysis

HepG2 cells were seeded in 6-well plates. After incubation for 24 hours, the cells were treated with different concentrations of drugs. Subsequently, the cells were stained using an Annexin V FITC/PI apoptosis detection kit (BD Biosciences, BD, USA). The apoptotic cells were detected on a Canto II flow cytometer (BD Biosciences, BD, USA). The same experiment was repeated three times.

### 3.13. Western Blotting

Cell proteins were extracted following treatment with corresponding drug concentrations. Then the protein concentration in the lysate was assessed using the BCA protein quantification kit. Proteins were separated on a 4 - 20% YoungPAGE precast gel electrophoresis, then transferred to polyvinylidene difluoride (PVDF) membranes. The membranes were blocked and incubated overnight at 4°C with antibodies, including anti-β-Tubulin (1:10000, AB0039, Abways, China), anti-c-Jun (1:1000, ET1608-3, HUABIO, China), anti-P-c-Jun (1:2000, ET1608-4, HUABIO), anti-c-Fos, and anti-P-c-Fos (1:500, ET1701-95, HUABIO). After washing thrice with TBST, the membranes were incubated with the secondary antibody for 1 hour at room temperature. Finally, the protein bands were detected and quantified by the ChemiDoc MP Imaging System and ImageJ software. The same experiment was repeated three times.

## 4. Results

### 4.1. Analysis of Frequency and Corresponding Syndromes of Traditional Chinese Medicines 

**Figure 1.**
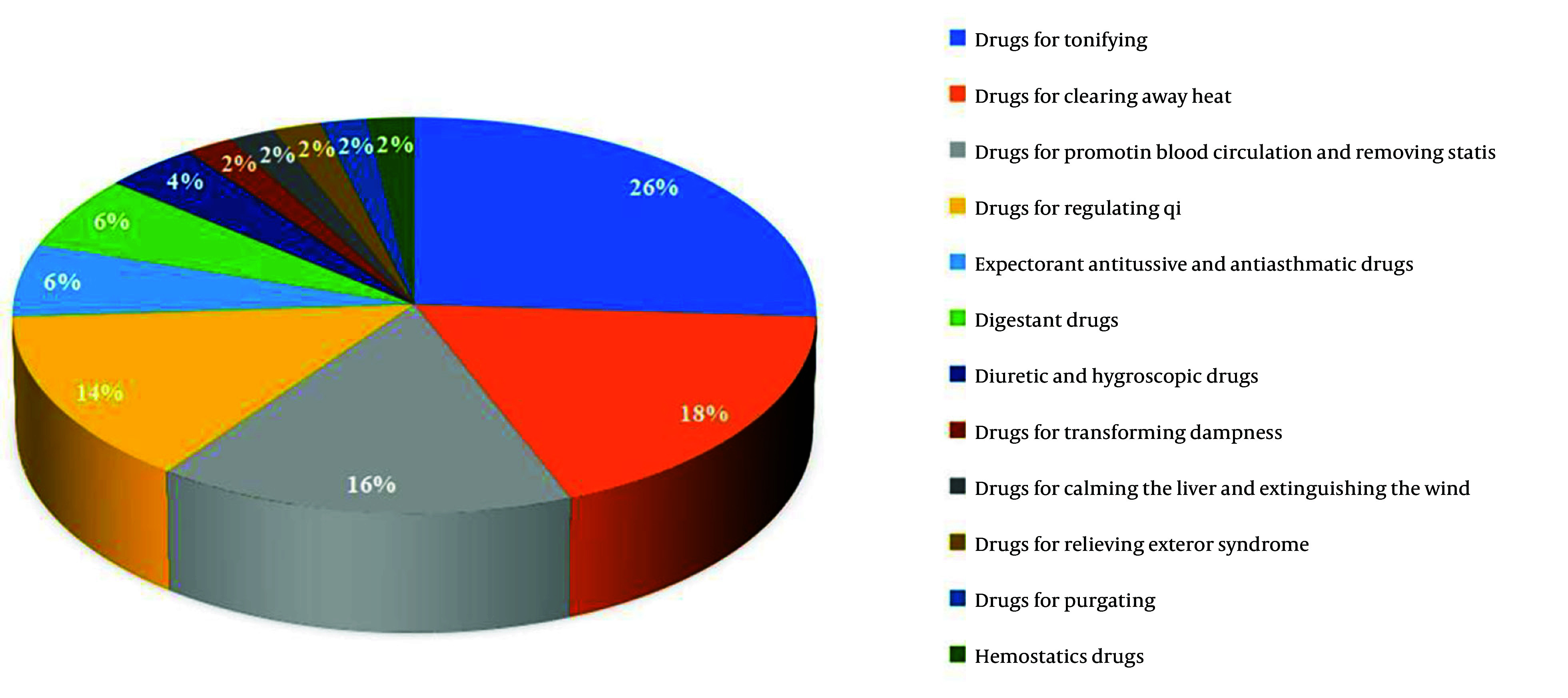
Classification of efficacy of high-frequency traditional Chinese medicine

A total of 107 eligible literature reports were selected, involving 107 prescriptions and 217 drugs. High-frequency drugs were defined as those administered more than six times, and a total of 50 high-frequency drugs were identified (Appendix 1 in Supplementary File). Among these, there are 13 types of invigorating qi drugs, such as *A. macrocephala* koidz, *A. membranaceus*, and *Ligustrum lucidum*. There are also nine types of heat-clearing medicines, like *Hedyotis diffusa*, *Scutellaria barbata*, and *Cremastra appendiculata*. Eight kinds of drugs for promoting blood circulation and removing stasis accounted for 16%, and seven kinds of medicines for regulating qi accounted for 14%. Invigorating qi and heat-clearing drugs were the most frequently employed, accounting for 44% of the overall frequency, indicating that the currently available TCMs for the treatment of liver cancer primarily target invigorating qi and heat-clearing drugs (Figure 1). Finally, a new compatible drug combination, including A*. macrocephala* koidz, *A. membranaceus*, *S. barbata*, and *C. appendiculata* (AASC), was chosen to investigate their potential synergistic mechanism against liver cancer.

### 4.2. Active Components and Targets Acquisition and Screening

**Figure 2.**
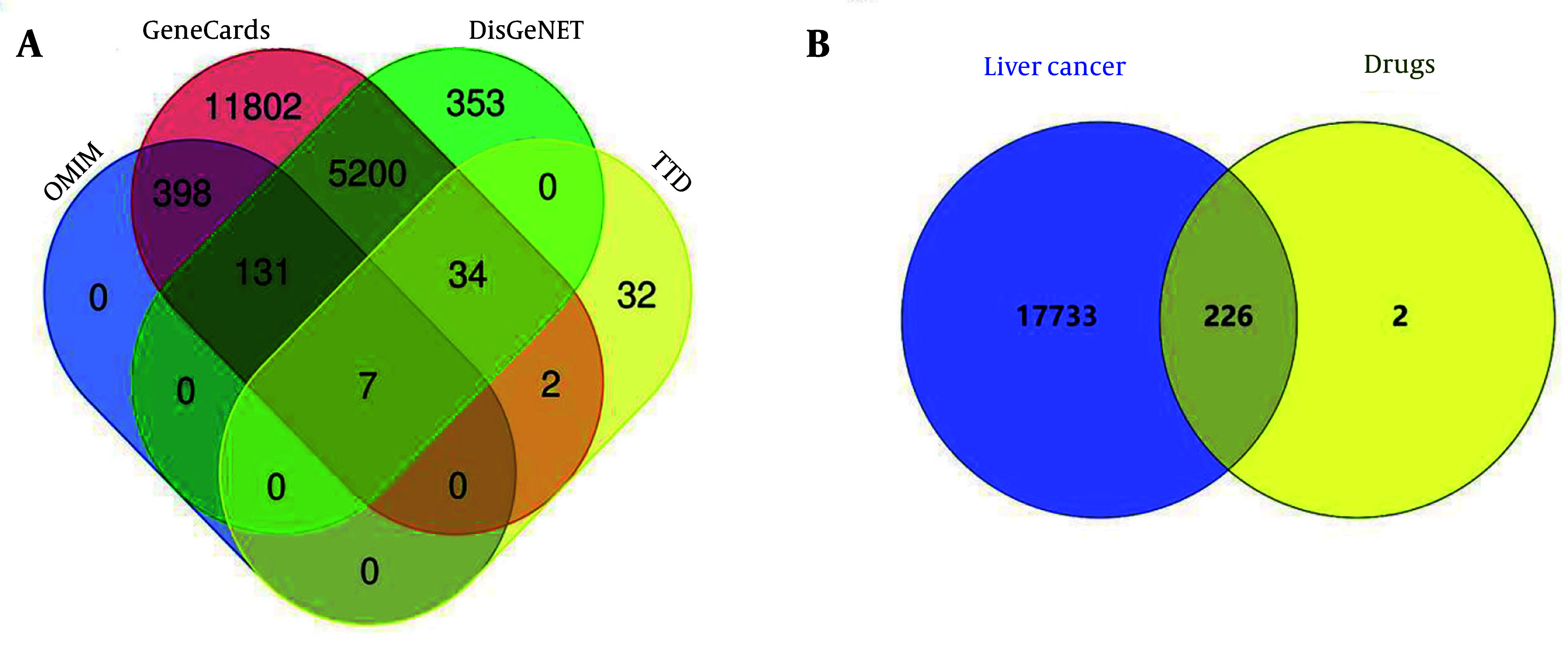
Venn diagram. A, Venn diagram of liver cancer targets; B, Venn diagram of common targets of drugs and liver cancer.

The results revealed 228 target genes associated with 48 active components of *A. macrocephala* koidz, *A. membranaceus*, *S. barbata*, and *C. appendiculata*. Detailed information about these 48 active components is provided in Appendix 2 in Supplementary File. A total of 17,689 liver cancer-related targets were obtained from disease databases (Figure 2A). Mapping identified 226 common targets as the action targets of AASC in liver cancer treatment (Figure 2B). 

### 4.3. "Traditional Chinese Medicine-Active Components-Target-Disease" Network and Analysis

**Figure 3.**
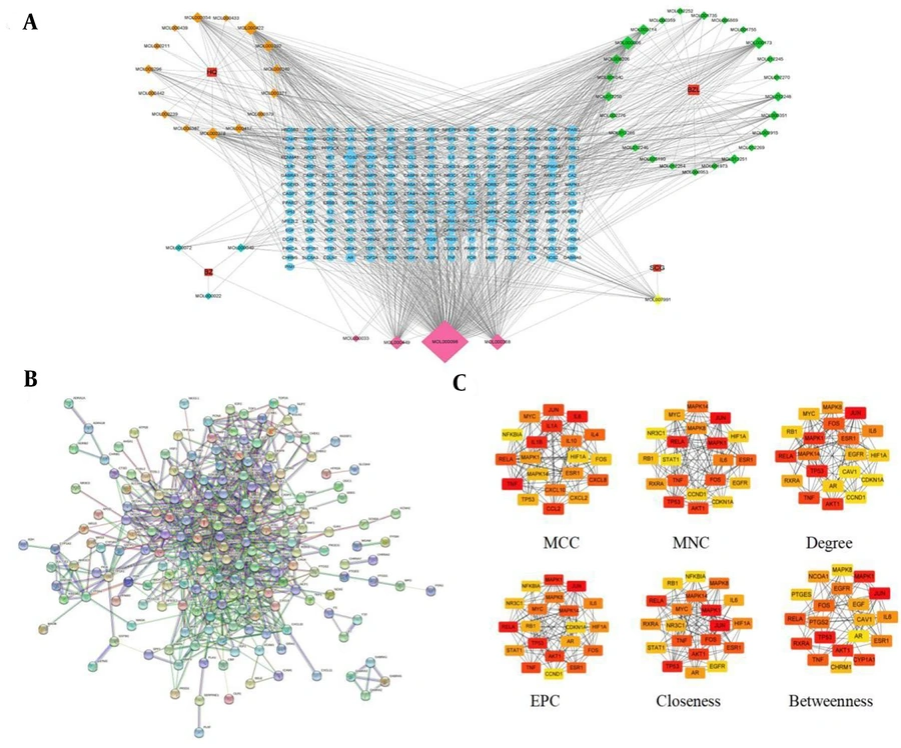
Network diagram based on the common targets. A, networks of "traditional Chinese Medicine-active components-targets-disease" were constructed using Cytoscape. HQ represents in the figure *Astragalus membranaceus*, BZ represents *Atractylodes macrocephala* koidz, BZL represents *Scutellaria barbata*, SCG represents *Cremastra appendiculata*. The rectangular nodes represent the drugs, the diamond nodes represent the active components of the drugs, the pink diamond nodes represent the common active components and the circular nodes represent the common targets of the drugs and liver cancer. The higher degree value of the node, the larger corresponding area, and the more important it is in the network. B, protein-protein interaction (PPI) network of common targets; C, PPI network topology analysis.

The network comprised 278 nodes and 1,131 edges, including four herbs, 48 active components, and 226 common targets (Figure 3A). By selecting the top 20 ranked nodes in each algorithm and identifying overlaps, we found that the top five compounds — quercetin, beta-sitosterol, kaempferol, stigmasterol, and luteolin — were repeatedly ranked highest and identified as the core components (Appendix 3 in Supplementary File). Additionally, all compounds showed high intestinal absorption and moderate total clearance rates (Appendix 4 in Supplementary File). None of them crossed the blood-brain barrier. They were all predicted to be non-Ames toxic and non-hepatotoxic. These features indicate strong potential of the selected compounds as lead candidates for HCC treatment. To identify the most influential targets, 226 common targets were uploaded to the STRING database platform, yielding a PPI network with 186 nodes and 817 edges (Figure 3B). Then, a topological analysis was conducted, and the results showed that JUN, MAPK1, RELA, TNF, ESR1, IL-6, TP53, and FOS were potential core targets of AASC against liver cancer (Figure 3C). 

### 4.4. Gene Ontology and Kyoto Encyclopedia of Genes and Genome Enrichment Analysis

**Figure 4.**
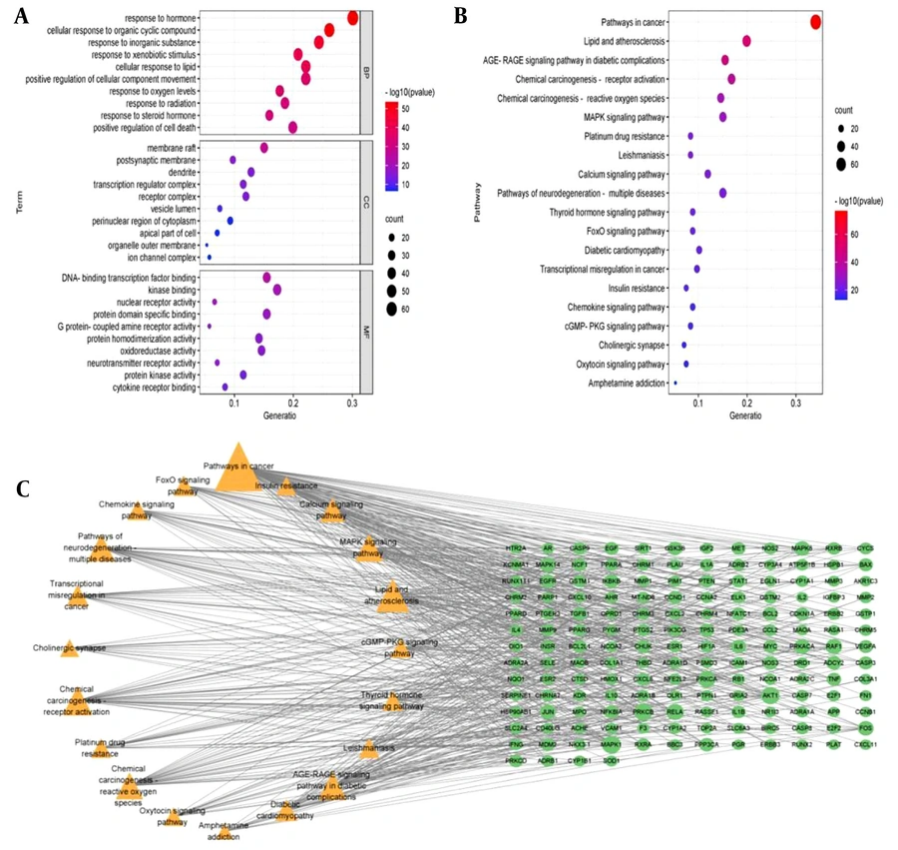
Gene Ontology (GO) and Kyoto Encyclopedia of Genes and Genome (KEGG) enrichment analysis. A, bubble diagram of GO analysis. All GO terms can be clustered into three groups: Biological process (BP); cellular component (CC) and molecular function (MF); B, bubble diagram of KEGG analysis; C, "Target-pathway" networks.

To further clarify the potential mechanisms of 226 targets, GO and KEGG enrichment analyses were performed. Based on gene count and adjusted P-value, the top three pathways in the biological process (BP) were response to hormone, cellular response to organic cyclic compound, and response to inorganic substance. The cellular component (CC) was primarily associated with membrane raft, postsynaptic membrane, and dendrite. In terms of molecular function (MF), the intersection targets were significantly enriched in activities like DNA-binding transcription factor binding, kinase binding, and nuclear receptor activity (Figure 4A). The KEGG pathway enrichment analysis showed that these genes were mainly involved in pathways in cancer, lipid and atherosclerosis, AGE-RAGE signaling pathway, and MAPK signaling pathway (Figure 4B). The top 20 pathways were then correlated with core targets to construct a "target-pathway" network diagram. The results revealed that the core targets were all enriched across multiple signaling pathways, suggesting the characteristics of multi-pathway and multi-target of AASC in the treatment of liver cancer (Figure 4C). 

### 4.5. Molecular Docking Analysis

**Figure 5.**
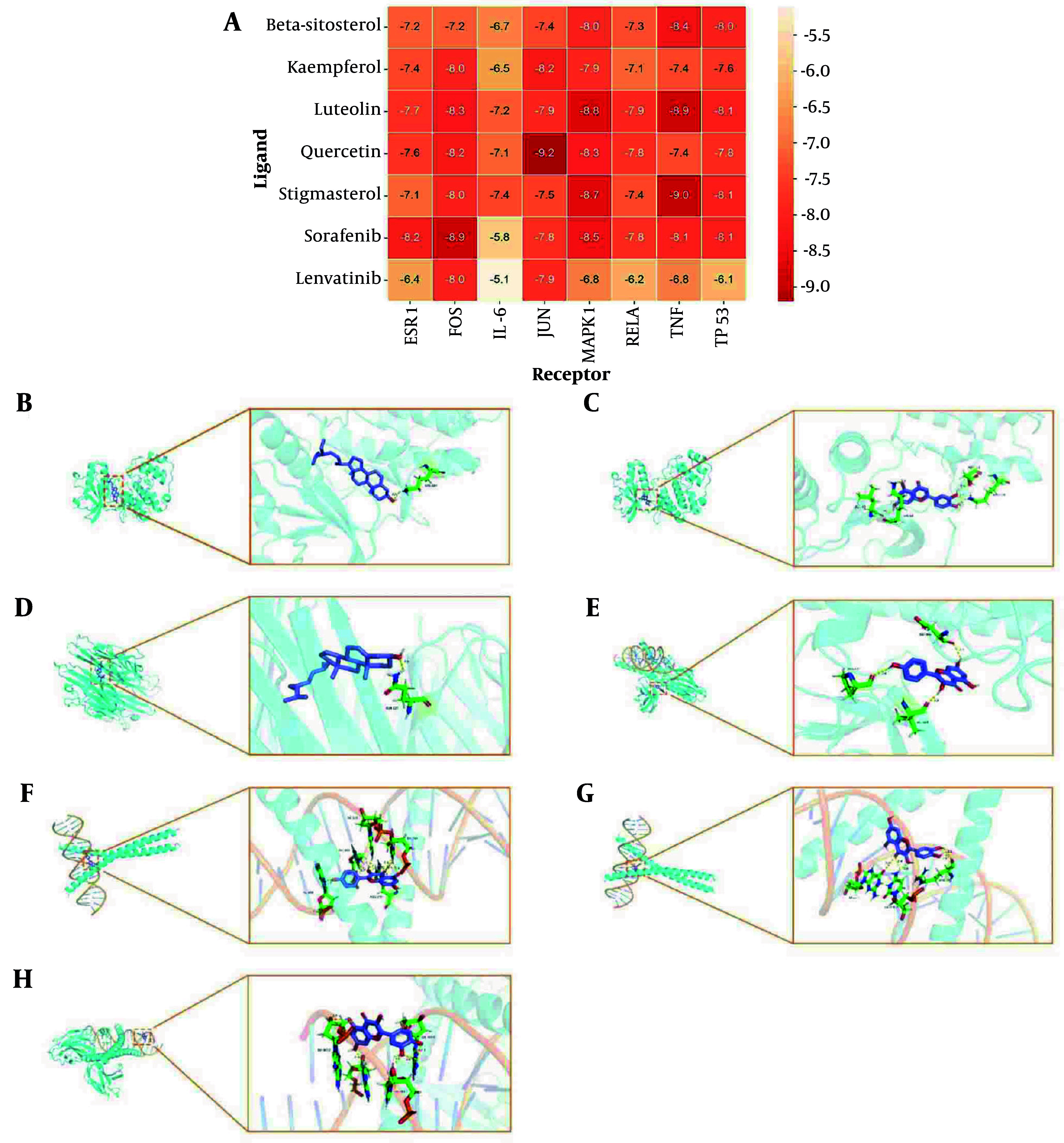
Molecular docking. A, heat map of molecular docking; B, beta-sitosterol-MAPK1 molecular docking pattern diagram; C, luteolin-MAPK1 molecular docking pattern diagram; D, stigmasterol-TNF molecular docking pattern diagram; E, kaempferol-FOS molecular docking pattern diagram; F, kaempferol-JUN molecular docking pattern diagram; G, quercetin-JUN molecular docking pattern diagram; H, quercetin-FOS molecular docking pattern diagram.

To validate the reliability of the network analysis, molecular docking was performed between the top five core components and eight key targets, with sorafenib and lenvatinib included as reference compounds commonly used in the clinical treatment of liver cancer. As shown in Figure 5A, all five core components exhibited binding energies below -6.0 kcal/mol across the selected targets, indicating strong ligand-receptor interactions. Notably, quercetin showed the most favorable binding profile, achieving a binding energy of -9.2 kcal/mol to JUN, which was lower than that of both sorafenib and lenvatinib under the same docking conditions. The molecular docking pattern diagrams showed the docking results of the compounds with the best binding affinity for each core target protein (Figure 5B-H). Quercetin, as the highest-degree core component, formed strong hydrogen bonds with JUN residues DG-211, DC-310, and ARG-270. It also exhibited a strong interaction with FOS, with a binding energy of -8.2 kcal/mol and hydrogen bond formation involving DG-5007, DA-4017, DA-5005, and DA-4019.

### 4.6. Quercetin Inhibits Migration and Promotes Apoptosis in HepG2 Cells

**Figure 6.**
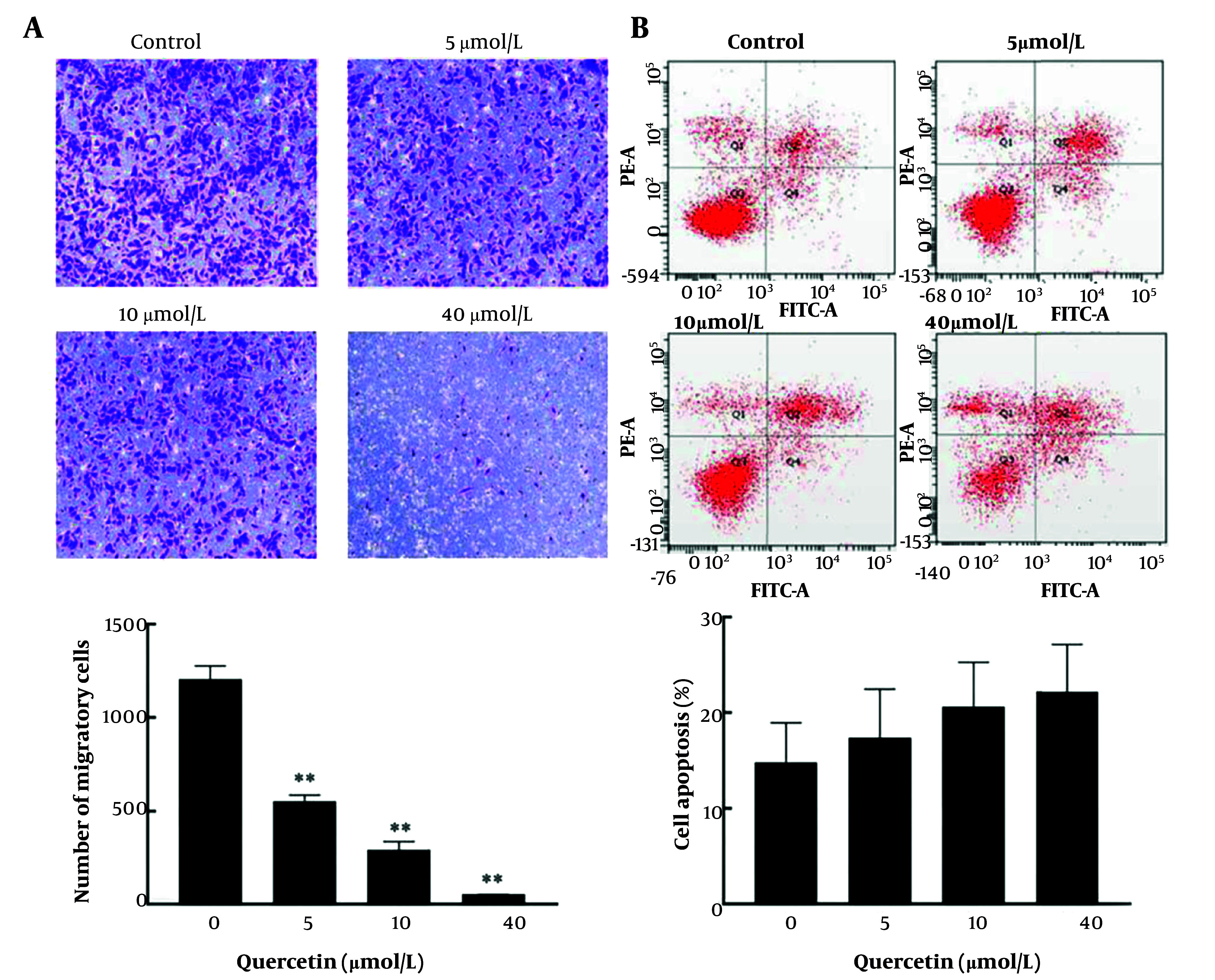
Inhibition of HepG2 cell migration and promotion of HepG2 apoptosis by quercetin. A, HepG2 cell migration. Compared to the control group, ** P < 0.01; B, HepG2 cell apoptosis.

Based on network pharmacological analysis, we selected quercetin, a key core component, to investigate its regulatory effect on liver cancer cells. After three repeated experiments, compared to the control group, quercetin treatment significantly reduced the number of migrated cells in a dose-dependent manner (Figure 6A). Additionally, although the differences were not statistically significant, a dose-dependent trend toward an increased percentage of apoptotic cells was observed in the treated group compared to the control group (Figure 6B). 

### 4.7. Quercetin Affects the Expression of Core Proteins

**Figure 7.**
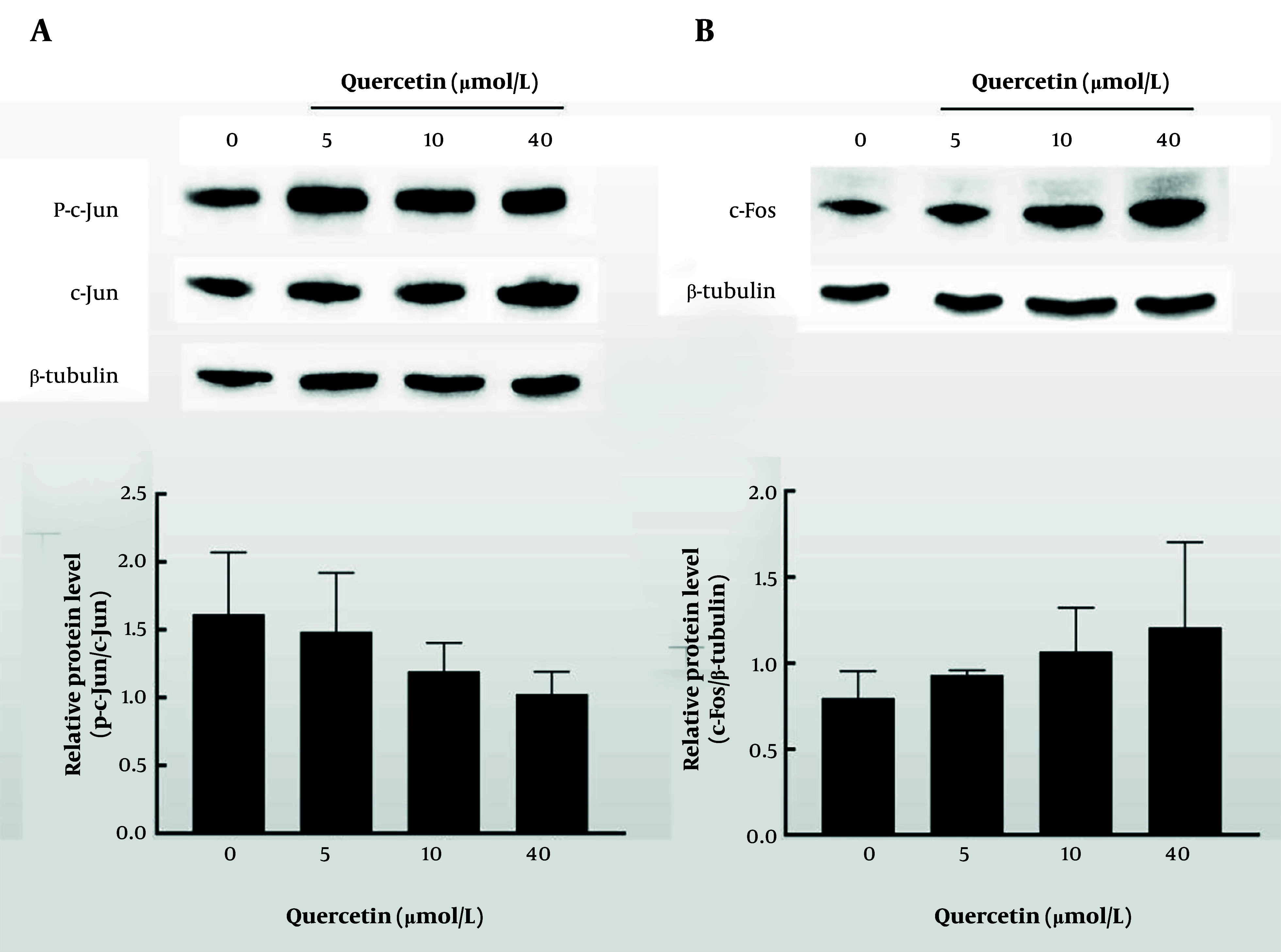
Effect of quercetin on core protein expression. A, the expression level of P-c-Jun/c-Jun was performed by western blotting; B, the expression level of c-Fos was performed by western blotting.

To further demonstrate the anti-liver cancer mechanism of quercetin, the expression levels of c-Jun and c-Fos were determined. In Figure 7A and B, the results of three repeated experiments suggested that quercetin treatment tended to reduce the expression of c-Jun while increasing the expression of c-Fos compared to the control group, although the changes were not statistically significant.

## 5. Discussion

Liver cancer, a highly malignant tumor with a global prevalence, presents challenges in effective treatment due to its high metastasis, recurrence, and chemical resistance rate (12). As a comprehensive approach to treating malignant tumors, TCMs offer advantages such as enhancing immunity, improving patient quality of life, and extending survival (13). Although TCMs play a dynamic role in the clinical management of liver cancer, uncertainties persist in the medication patterns and mechanisms through which high-frequency drug combinations counteract liver cancer occurrence and development.

In this study, we systematically summarized prescription compositions and medication rules from prior research on the treatment of liver cancer using TCMs. After analyzing 107 prescriptions, 50 high-frequency TCMs were obtained, which were dominated by qi-invigorating herbs (such as *A. macrocephala* koidz, *A. membranaceus*, *L. lucidum*) and heat-clearing herbs (such as *H. diffusa*, *S. barbata*, *C. appendiculata*). These findings align with the Chinese medicine treatment of liver cancer, which focuses on strengthening the spleen, invigorating qi, and detoxifying and dispersing nodules (14-16). Both *A. macrocephala* koidz and A*. membranaceus* are vital for invigorating qi. The former tonifies spleen and stomach meridians (17), while the latter nourishes spleen and lung meridians, invigorates qi, promotes fluid production, and nourishes blood (18). These two herbs complement each other effectively. Furthermore, *S. barbata* and *C. appendiculata* are crucial heat-clearing drugs. *S. barbata* has the effects of clearing heat and detoxifying, resolving blood stasis, and diuresis (19). *Cremastra appendiculata* belongs to the liver and spleen meridians, which has the effects of clearing heat, detoxifying, softening, and dispersing nodules (20). The four drugs of *A. macrocephala* koidz, *A. membranaceus*, *S. barbata*, and *C. appendiculata* are not only a combination of invigorating qi and heat-clearing drugs but also the core components of many formulas, which are widely used in clinical practice for tumor treatment.

According to network pharmacology analysis, the construction of a "traditional Chinese medicine-active components-target-disease" network diagram identified five core active components. These components, primarily flavonoids and phytosterols, include quercetin, beta-sitosterol, kaempferol, stigmasterol, and luteolin. Flavonoids are known to be potential anticancer agents with an excellent safety profile (21). Quercetin, a plant-derived polyphenol, exhibits diverse biological actions, such as anti-carcinogenic, anti-inflammatory, antioxidant, and antiviral effects (22). It mainly inhibits liver cancer progression by regulating apoptosis, migration, invasion, cell cycle arrest, and autophagy (23-25). Therefore, a growing body of research demonstrating quercetin’s antitumor properties places it as a prospective anti-cancer agent, not only as a stand-alone therapy but also as a means of enhancing currently available therapeutic choices for advanced HCC. Abdu et al. (26) confirmed that quercetin alone or in combination with sorafenib (the first approved drug for the treatment of advanced HCC and continuing to be the gold standard therapy for HCC) significantly inhibited HCC growth, induced cell cycle arrest, and induced apoptosis and necrosis. Similarly, luteolin exhibits various biological effects like anti-inflammatory, anti-allergic, and anti-cancer, capable of preventing and treating numerous cancer types (27). Published reports indicate that kaempferol promoted cell apoptosis in a dose-dependent manner, inhibited HepG2 cell proliferation, and the inhibition rate increased with the increase in drug concentration and incubation time (28). In recent years, mounting evidence supports the broad anti-carcinogenic effects of phytosterols. They’ve been linked to the mitigation of breast, prostate, lung, liver, stomach, and ovarian cancers. Kim et al. (29) revealed that stigmasterol could induce apoptosis in HepG2 cells by up-regulating the expression of pro-apoptotic genes (Bax, p53) and down-regulating the anti-apoptotic genes (Bcl-2). These findings suggest quercetin, beta-sitosterol, kaempferol, stigmasterol, and luteolin may be good anti-carcinogenic components.

To further explore the binding activity between core components and core targets, and verify network pharmacology findings, molecular docking was performed. The results showed that quercetin had strong binding energy with JUN and FOS. As reported by Chen et al., quercetin could suppress TNF-α induced apoptosis and inflammation by blocking NF-κB and AP-1 signaling pathways (30). Besides, Hsieh et al. (31) revealed that quercetin inhibits TNF-α-induced cell migration by reducing MMP-9 expression via c-Fos and NF-κB pathways. To further verify the relationship between quercetin and the transcription factors c-Fos and c-Jun, in vitro experiments were performed. These results were consistent with network pharmacology prediction, indicating that quercetin induced apoptosis and inhibited migration of HepG2 cells by affecting c-Jun and c-Fos protein expressions. Moreover, docking results showed that quercetin exhibited comparable or even stronger binding affinities than sorafenib and lenvatinib across multiple targets, further supporting its potential as a promising anticancer compound.

Taken together, this study fully demonstrated the multi-component, multi-pathway, and multi-target characteristics of *A. macrocephala* koidz, *A. membranaceus*, *S. barbata*, and *C. appendiculata* in liver cancer treatment, providing a foundational basis for future clinical applications. Nonetheless, there are still some limitations. Firstly, the public online databases used are imperfect and constantly updated, so some active components of drugs and their target genes may not be included in the analysis. Secondly, although certain trends (such as changes in migration capacity and apoptosis rate) were observed in the in vitro experiments, some of the results did not reach statistical significance. Therefore, future studies should further validate these findings through in vivo experiments and optimize the experimental design to better evaluate the therapeutic effects of quercetin in liver cancer. Despite these limitations, this study provides powerful tools and preliminary data for further research on TCMs in liver cancer.

ijpr-24-1-162305-s001.pdf

## Data Availability

The data presented in this study are uploaded during submission as a supplementary file and are openly available for readers upon request.
