# Supplementary material for: Integrating Network Pharmacology and Experimental Validation to Identifying Key Herbal Components and Targets for Liver Cancer
Source: Iran J Pharm Res. 2025 Sep 3;24(1):e162305. doi: 10.5812/ijpr-162305 (PMC12524083; doi:10.5812/ijpr-162305)
Supplement: ijpr-24-1-162305-s001.pdf [file ijpr-24-1-162305-s001.pdf]

Appendix 1: Summary of high-frequency traditional Chinese medicine

Medicine	Frequency	Medicine Frequency
Atractylodes macrocephala koidz	62	Drugs for tonifying
Poria cocos	58	Diuretic and hygroscopic drugs
Astragalus membranaceus	57	Drugs for tonifying
Licorice	53	Drugs for tonifying
Hedyotis diffusa	49	Drugs for clearing away heat
Radix bupleuri	48	Drugs for relieving exterior syndrome
White peony	43	Drugs for tonifying
Scutellaria barbata	36	Drugs for clearing away heat
Codonopsis pilosula	33	Drugs for tonifying
Angelica sinensis	30	Drugs for tonifying
Pericarpium citri reticulatae	29	Drugs for regulating qi
Curcuma zedoary	28	Drugs for promoting blood circulation and removing stasis
Radix curcumae	27	Drugs for promoting blood circulation and removing stasis
Salvia miltiorrhiza	26	Drugs for promoting blood circulation and removing stasis
Fructus aurantii	22	Drugs for regulating qi
Pinelliae rhizoma	20	Expectorant antitussive and antiasthmatic drugs
Artemisiae scopariae herba	20	Drugs for clearing away heat
Coix seed	19	Diuretic and hygroscopic drugs
Turtle shell	18	Drugs for tonifying
Red peony	18	Drugs for clearing away heat
Yam	17	Drugs for tonifying
Hawthorn	17	Digestant drugs
Scutellaria baicalensis	16	Drugs for clearing away heat
Medicine	Frequency	Medicine Frequency
Ligusticum wallichii	14	Drugs for promoting blood circulation

		and removing stasis
Rehmannia glutinosa	14	Drugs for clearing away heat
Chinese wolfberry	14	Drugs for tonifying
Fructus ligustri lucidi	13	Drugs for tonifying
Pseudostellariae radix	13	Expectorant antitussive and antiasthmatic drugs
Corydalis rhizoma	13	Drugs for promoting blood circulation and removing stasis
Persicae semen	12	Drugs for promoting blood circulation and removing stasis
Oysters	11	Drugs for calming the liver and extinguishing the wind
Sand ginseng	11	Drugs for tonifying
Hordei fructus germinatus	10	Drugs for regulating qi
Panax ginseng C. A. Mey	10	Drugs for tonifying
Divine qu	10	Digestant drugs
Gardenia	10	Drugs for clearing away heat
Rheum officinale	9	Drugs for purgating
Chinaberry	8	Drugs for regulating qi
Membranes of chicken gizzards	8	Digestant drugs
Amomum villosum	8	Drugs for transforming dampness
Cyperus rotundus	8	Drugs for regulating qi
Safflower	7	Drugs for promoting blood circulation and removing stasis
Ophiopogon japonicus	7	Drugs for tonifying
Pseudo-ginseng	7	Hemostatics drugs
Aurantii fructus immaturus	7	Drugs for regulating qi
Fructus aurantii	7	Expectorant antitussive and antiasthmatic drugs

Medicine	Frequency	Medicine Frequency
Cremastra appendiculata	6	Drugs for clearing away heat

Lobeliae chinensis herba	6	Drugs for clearing away heat
Aucklandiae radix	6	Drugs for regulating qi
Sparganii rhizoma	6	Drugs for promoting blood circulation and removing stasis

Appendix 2: Effective components of Atractylodes macrocephala koidz, Astragal

us membranaceus, Scutellaria barbata and Cremastra appendiculata

Number	Mol ID	Active Components	OB (%)	DL	Source
1	MOL000033	(3S,8S,9S,10R,13R,14S,17R)-10,13-dimethyl-17-[(2R,5S)-5-propan-2-yloctan-2-yl]-2,3,4,7,8,9,11,12,14,15,16,17-dodecahydro-1H-Cyclopenta[a]phenanthren-3-ol	36.23	0.78	Astragalus membranaceus、Atractylodes macrocephala koidz
2	MOL000098	quercetin	46.43	0.28	Astragalus membranaceus、Scutellaria barbata
3	MOL000358	beta-sitosterol	36.91	0.75	Scutellaria barbata、Cremastra appendiculata
4	MOL000449	Stigmasterol	43.83	0.76	Scutellaria barbata、Cremastra appendiculata
5	MOL000211	Mairin	55.38	0.78	Astragalus membranaceus
6	MOL000239	Jaranol	50.83	0.29	Astragalus membranaceus
7	MOL000296	hederagenin	36.91	0.75	Astragalus membranaceus
8	MOL000354	isorhamnetin	49.60	0.31	Astragalus membranaceus
9	MOL000371	3,9-di-O-methylnissolin	53.74	0.48	Astragalus membranaceus
10	MOL000378	7-O-methylisomucronulatol	74.69	0.30	Astragalus membranaceus
11	MOL000379	9,10-dimethoxypterocarpan-3-O-β-D-glucoside	36.74	0.92	Astragalus membranaceus

Number	Mol ID	Active Components	OB (%)	DL	Source
12	MOL000380	(6aR,11aR)-9,10-dimethoxy-6a,11a-dihydro-6H-benzofurano[3,2-c]chromen-3-ol	64.26	0.42	Astragalus membranaceus
13	MOL000387	Bifendate	31.10	0.67	Astragalus membranaceus
14	MOL000392	formononetin	69.67	0.21	Astragalus membranaceus
15	MOL000417	Calycosin	47.75	0.24	Astragalus membranaceus
16	MOL000422	kaempferol	41.88	0.24	Astragalus membranaceus
17	MOL000433	FA	68.96	0.71	Astragalus membranaceus
18	MOL000439	isomucronulatol-7,2'-di-O-glucosiole	49.28	0.62	Astragalus membranaceus
19	MOL000442	1,7-Dihydroxy-3,9-dimethoxy pterocarpene	39.05	0.48	Astragalus membranaceus
20	MOL000022	14-acetyl-12-senecioyl-2E,8Z,10E-atractylentriol	63.37	0.30	Atractylodes macrocephala koidz
21	MOL000049	3β-acetoxyatractylone	54.07	0.22	Atractylodes macrocephala koidz
22	MOL000072	8β-ethoxy atractylenolide Ⅲ	35.95	0.21	Atractylodes macrocephala koidz
23	MOL001040	(2R)-5,7-dihydroxy-2-(4-hydroxyphenyl)chroman-4-one	42.36	0.21	Scutellaria barbata
24	MOL012245	5,7,4'-trihydroxy-6-methoxyflavanone	36.63	0.27	Scutellaria barbata
25	MOL012246	5,7,4'-trihydroxy-8-methoxyflavanone	74.24	0.26	Scutellaria barbata
26	MOL012248	1. hydroxy-7,8-dimethoxy-2-(4-methoxyph	65.82	0.33	Scutellaria barbata

		enyl)chromone			
27	MOL012250	7-hydroxy-5,8-dimethoxy-2-phenyl-chromone	43.72	0.25	Scutellaria barbata
28	MOL012251	Chrysin-5-methylether	37.27	0.20	Scutellaria barbata
29	MOL012252	9,19-cyclolanost-24-en-3-ol	38.69	0.78	Scutellaria barbata
30	MOL002776	Baicalin	40.12	0.75	Scutellaria barbata
31	MOL012254	campesterol	37.58	0.71	Scutellaria barbata
32	MOL000953	CLR	37.87	0.68	Scutellaria barbata
33	MOL012266	rivularin	37.94	0.37	Scutellaria barbata
34	MOL001973	Sitosteryl acetate	40.39	0.85	Scutellaria barbata
35	MOL012269	Stigmasta-5,22-dien-3-ol-acetate	46.44	0.86	Scutellaria barbata
36	MOL012270	Stigmastan-3,5,22-triene	45.03	0.71	Scutellaria barbata
37	MOL000173	wogonin	30.68	0.23	Scutellaria barbata
38	MOL001735	Dinatin	30.97	0.27	Scutellaria barbata
39	MOL001755	24-Ethylcholest-4-en-3-one	36.08	0.76	Scutellaria barbata
40	MOL002714	baicalein	33.52	0.21	Scutellaria barbata
41	MOL002915	Salvigenin	49.07	0.33	Scutellaria barbata
42	MOL000351	Rhamnazin	47.14	0.34	Scutellaria barbata
43	MOL000359	sitosterol	36.91	0.75	Scutellaria barbata
44	MOL005190	eriodictyol	71.79	0.24	Scutellaria barbata
45	MOL005869	daucostero_qt	36.91	0.75	Scutellaria barbata
46	MOL000006	luteolin	36.16	0.25	Scutellaria barbata

Number	Mol ID	Active Components	OB (%)	DL	Source
47	MOL008206	Moslosooflavone	44.09	0.25	Scutellaria barbata
48	MOL007991	2-methoxy-9,10-dihydrophenanthrene-4,5-diol	44.97	0.18	Cremastra appendiculata

Appendix 3:Algorithm analysis of effective components

score	CytoHubba analysis			
	Betweenness	EPC	MCC	Degree
1	MOL000098	MOL000098	MOL000098	MOL000098
2	MOL000006	MOL000358	MOL000422	MOL000358
3	MOL000358	PTGS1	MOL000006	MOL000422
4	MOL000422	PTGS2	MOL000173	MOL000449
5	MOL002714	MOL000449	MOL000378	MOL000006
6	MOL000449	MOL000422	MOL000358	MOL000173
7	PTGS2	HSP90AB1	MOL000392	MOL000378
8	BZL	MOL000378	PTGS2	PTGS2
9	MOL000173	MOL000173	MOL002714	MOL000392
10	MOL000392	NCOA2	MOL000354	MOL002714
11	MOL000378	MOL000006	MOL000449	MOL000354
12	NCOA2	PRSS1	PTGS1	PTGS1
13	PTGS1	PRKACA	MOL007991	HSP90AB1
14	MOL000354	MOL000392	BZL	MOL007991
15	HSP90AB1	MOL000354	HSP90AB1	NCOA2
16	PRKACA	ADRB2	NCOA2	BZL
17	HQ	DPP4	MOL012248	PRSS1
18	PRSS1	SCN5A	MOL012250	PRKACA
19	PPARG	MOL007991	MOL012251	MOL012248
20	DPP4	AR	MOL008206	MOL012250

Appendix 4: ADMET Parameters

ADMET Parameters	Phytochemicals				
	**quercetin**	**β -Sitosterol**	**kaempferol**	**stigmasterol**	**luteolin**
Absorption and distribution					
BBB	No	No	No		
Intestinal absorption (%)	77.21	94.46	74.29	94.97	81.13
PGS	Yes	No	Yes	No	Yes
PGI	No	No	No	Yes	No
Metabolism					
CYP3A4 substrate	No	Yes	No	Yes	No
CYP2D6 substrate	No	No	No	No	No
CYP3A4 inhibition	No	No	No	No	No
CYP2C9 inhibition	No	No	No	No	YES
CYP2C19 inhibition	No	No	No	No	No
CYP2D6 inhibition	No	No	No	No	No
CYP1A2 inhibition	YES	No	YES	No	YES
Excretion					
Total Clearance (log mL/min/kg)	0.407	0.628	0.477	0.618	0.495
Toxicity					
AMES Toxicity	No	No	No	No	No
Hepatotoxicity	No	No	No	No	No
Skin Sensitization	No	No	No	No	No
